# A comparison of EQ-5D index scores using the UK, US, and Japan preference weights in a Thai sample with type 2 diabetes

**DOI:** 10.1186/1477-7525-6-71

**Published:** 2008-09-23

**Authors:** Phantipa Sakthong, Rungpetch Charoenvisuthiwongs, Rossamalin Shabunthom

**Affiliations:** 1 Department of Pharmacy Practice, Faculty of Pharmaceutical Sciences, Chulalongkorn University, Bangkok, Thailand; 2 Department of Pharmacy Administration, Faculty of Pharmaceutical Sciences, Chulalongkorn University, Bangkok, Thailand; 3 Sawangdandin Crown Prince Hospital, Sakolnakorn, Thailand

## Abstract

**Background:**

Data are scarce on the comparison of EQ-5D index scores using the UK, US, and Japan preference weights in other populations. This study was aimed to examine the differences and agreements between these three weights, psychometric properties including test-retest reliability, convergent and known-groups validity, and the impact of differences in the EQ-5D scores on the outcome of cost-utility analysis in Thai people.

**Methods:**

A convenience sample of 303 type 2 diabetic outpatients (18 years or older) from a cross-sectional study was examined. ANOVA and pos-hoc Bonferroni tests were used to determine the differences among the three EQ-5D scores. The agreements among the EQ-5D scores were assessed employing intraclass correlations coefficients (ICCs) and Bland-Altman plots. The ICCs were utilized to examine the test-retest reliability. Spearman's rho correlation coefficients were used to assess the convergent validity between the EQ-5D scores and sociodemographic & clinical data, and health status. Mann-Whitney U tests were used to test the differences in EQ-5D scores between the known groups including HbA1c level (cut point of 7%), and the presence of diabetic complications namely neuropathy, retinopathy, nephropathy and cardiovascular diseases. Seven hypothetical decision trees were created to evaluate the impact of differences in the EQ-5D scores on the incremental cost-utility ratio (ICUR).

**Results:**

The US weights yielded higher scores than those of the UK and the Japan weights (*p *< 0.001, both), while the UK and the Japan weighted scores did not differ (*p *> 0.05). Both UK and US scores had more agreement with each other than with the Japan scores. Regarding psychometric properties, the Japan scheme provided better test-retest reliability, convergent and known-groups validity than both UK and US schemes. The variation in EQ-5D scores estimated from UK, US, and Japan preference weights had a marginal impact on ICUR (range: 1.23–6.32%).

**Conclusion:**

Since the Japan model showed more preferable psychometric properties than the UK and the US models and the differences in these EQ-5D scores had a small impact on ICUR, we recommended that for both clinical and policy purposes the Japan scheme should be used in Thai people. However, more research needs to be done.

## Background

The health utility (HU) approach to assessing health-related quality of life (HRQoL) is a commonly used technique for determining preferences for health outcomes in evaluation of public health and healthcare interventions such as cost-utility analyses (CUA) [1,2]. In CUA, a utility score is assigned to the health state on the cardinal scale in which dead = 0 and perfect health = 1 to indicate their preferences for different outcomes. The utility score is incorporated into quality-adjusted life-year (QALYs) which combine, in a single index, gains or losses in quantity (life expectancy) and quality of life (HU). The EuroQoL (EQ-5D) is the most frequently used HU instrument for calculating QALYs based on actual measurements of patients' HRQoL [3].

The EQ-5D instrument consists of a five-item descriptive system of health states and a visual analog scale (VAS). Scores for the five health states can be converted into a utility index score by using scores from value sets (preference weights) elicited from a general population. The best-known preference weights were derived from samples of the United Kingdom (UK) population which is the original one for estimating EQ-5D index scores [4]. The UK-based preference weights are applied to other populations when country-specific weights are not available. However, evidence suggests valuations of health states could differ for people in different countries due to differences in demographic backgrounds, social-cultural values, and economic systems [5-8]. Thus, it is advisable to use country-specific weights in a given country if available.

Unfortunately, preference weights of EQ-5D for Thai people are not available yet. Valuation of the EQ-5D health states nationwide is a complex, time-consuming, and expensive task, so applying other existing preference weights is essential if not available in the country. Nevertheless, whose weighting scheme or which cultural/country-specific populations are appropriate are not known for Thai population. Besides the UK weights, there are a number of other countries having their own population-based preference weights for the EQ-5D [7,9-14]. Of these, the United States (US) weight scheme is a unique D1 model [13] different from the UK model (N3 model) that was applied to other countries' models. Studies have also shown that EQ-5D scores derived from the US weights were different from those of the UK [15-17].

Japan has been the first Asian country to develop its own preference weights of EQ-5D since 2002 [11]. The Japan model was chosen to represent Asian preference weights. We were interested in knowing how different EQ-5D index scores using the UK, US, and Japan preference weights were. Little was also known about psychometric properties of these schemes in different cultural contexts and specific patient samples (all models were developed in general population). Therefore, we would like to determine the differences and agreements among these three countries' preference weighted scores (the three countries are located in three different continents as well) using a Thai patient sample. Their psychometric properties including test-retest reliability, convergent and known-groups validity were also explored. The psychometric properties would provide additional evidence of validity for the use of the EQ-5D index score in Thai settings. Moreover, we would examine the impact of differences in the EQ-5D scores on the outcome of CUA employing hypothetical scenarios.

## Methods

### Subjects and procedures

The data used in this paper was derived from a cross-sectional study [18]. In this study, a convenience sample of 303 type 2 diabetic outpatients was collected from the General Police Hospital in Bangkok, Thailand, between January-June, 2007. Patients with type 2 diabetics waiting for seeing physicians were approached to participate in this study. Patients who were eligible for the study were at least 18 years old and were able to understand the Thai language. Patients with health problems or cognitive impairments that could not complete interview were excluded. The face-to-face interviews include Morisky Medication Adherence Scale, Center for Epidemiologic Studies Depression (CES-D), EQ-5D questionnaire, VAS, sociodemographic and clinical data, together with reviewing medical records. In addition, about one-fifth of this sample (N = 64) was randomly selected to conduct one-two week test-retest reliability via telephone. This study was approved by the Ethics Committee of the Police Hospital.

### EQ-5D: UK, US, and Japan preference weights

The EQ-5D includes a five-item descriptive system, with one item for each of the following health attributes: mobility, self care, usual activity, pain/discomfort, and anxiety/depression. Each attribute has three levels: no problem, some problem, and major problem. A total of 243 possible health states are generated.

The UK valuation study was conducted based on the Measuring and Valuation Health (MVH) protocol to collect a general adult population in the United Kingdom (England, Scotland, and Wales) [4,19]. The preference values for 42 core health states were elicited using time trade-off (TTO) methods. The valuations of the 42 health states were then interpolated by regression models to predict the index scores for all EQ-5D possible health states. The UK model consists of a set of variables representing each EQ-5D health dimension, with two dummy variables representing the levels of each dimension. A dichotomous variable (N3) was also added to the model to indicate if level 3 (major problem) occurs within at least one dimension.

The US health state valuation study was derived based on the UK MVH protocol. But the US algorithm replaced the N3 variable by D1, which represents additional number of dimensions at level 2 and 3 beyond the first [13].

The Japan valuation study is a quasi-replication of the UK MVH protocol using the modified protocol, where each respondent was presented with 17 health states, instead of 42 health states. The plain main effects model was preferred [11].

### Data analysis

The EQ-5D index scores were calculated using the UK, US, and Japan preference weights. We first determined the differences among the three index scores using ANOVA, followed by pos-hoc Bonferroni tests. The agreements among the EQ-5D scores using the UK, US, and Japan preference weights were also assessed employing intraclass correlations coefficients (ICCs) and Bland-Altman plots [20]. We then examined the psychometric properties of these EQ-5D scores using the following approaches: one-two week test-retest reliability, convergent validity and known-groups validity [21].

To evaluate the test-retest reliability, intraclass correlations coefficients (ICCs) were employed. For convergent validity, we assessed the associations between the three EQ-5D scores and sociodemographic & clinical data and health status including age, gender, income, duration of diabetes, body mass index (an indicator of obesity), HbA1c level, number of diabetic complications, CES-D scores, and VAS scores using Spearman's rho correlation coefficients.

Concerning known-groups validity, we examined the ability of the three EQ-5D scores using the UK, US, and Japan preference weights to discriminate between clinical known groups including HbA1c level (below versus equal or above 7%), and presence and absence of diabetic complications namely neuropathy, retinopathy, nephropathy and cardiovascular. Mann-Whitney U tests were used to test the differences in EQ-5D index scores between these known groups because the distributions of EQ-5D utility scores had a number of outliers. All analyses were performed using SPSS version 13.0.

To evaluate the impact of the differences in EQ-5D index scores using UK, US, and Japan preference weights on CUA, seven hypothetical decision trees were created. We compared a new drug (Drug A) with an existing drug (Drug B). The details of each data component of the base-case scenario (decision tree 1) are presented in Table 1. We also created decision trees 2–7 which overestimated the base-case utility scores by mean and median differences in EQ-5D index scores between three pairs of preference weights: UK versus US, UK versus Japan, and US versus Japan, respectively. We computed the incremental cost-utility ratio (ICUR) which is equal to the ratio of incremental costs (cost of drug A minus cost of drug B) over incremental QALY (QALY of drug A minus QALY of drug B).

**Table 1:** Data component of the base-case scenario (decision tree 1)

	Drug A (new drug)		Drug B (existing drug)	
	
	Success	Failure	Success	Failure
Probability of treatment results	0.6	0.4	0.5	0.5
Survival (year)	10	5	8	4
Utility scores	0.9	0.5	0.8	0.4
Cost (Baht)	100,000	50,000	80,000	40,000

## Results

The sociodemographic and clinical characteristics are shown in Table 2. The mean age was 61.6 years old (SD: 11.4; range: 27–90) and 71% were female. The median of income was 5,000 Baht/month (interquartile: 0–16,300) (34 Baht ≈ 1 US$). The mean HbA1c, mean BMI (body mass index), and mean duration of diabetes (told by the patients) were 7.7% (SD: 1.7; range: 4.0–15.8) and 26.7 Kg/m^2 ^(SD: 1.7; range: 16.7–55.2), and 12.2 years (SD: 8.4; range: 0–50), respectively. Regarding diabetic complications, there were 124 cases (40.9%) of neuropathy, 51 (16.8%) of retinopathy, 25 (8.3%) of nephropathy, and 44 (14.5%) of cardiovascular disorders. The median CES-D scores were 5 (interquartile: 2–10). The mean VAS scores were 0.69 (SD: 0.16; range: 0.10–1.00).

**Table 2:** Characteristics of type 2 diabetic patients (N = 303)

**Characteristics**		**Value**
Age (year)	Mean ± SD	61.1 ± 11.4
	Range	27–90
Gender	Female	71%
Income (Baht per month)	Median (25^th ^percentile, 75^th ^percentile)	5,000 (0, 16,300)
Duration of diabetes (year)	Mean ± SD	12.2 ± 8.4
	Range	0–50
HbA1c level	Mean ± SD	7.7 ± 1.7%
	Range	4.0–15.8%
Body mass index (Kg/m^2^)	Mean ± SD	26.7 ± 1.7
	Range	16.7–55.2
Diabetic complication	Neuropathy	124 (40.9%)
	Retinopathy	51 (16.8%)
	Nephropathy	25 (8.3%)
	Cardiovascular disorders	44 (14.5%)
CES-D scores	Median (25^th ^percentile, 75^th ^percentile)	5 (2, 10)
VAS scores (usual scores: 0–1)	Mean ± SD	0.69 ± 0.16
	Range	0.10–1.00

**Figure 1:**
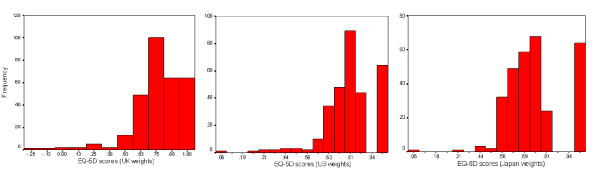
Distribution of EQ-5D scores derived from the UK, US, and Japan preference weights.

The distributions of EQ-5D scores derived from the UK, US, and Japan preference weights were skewed to the left (Figure 1). The mean (95% CI) EQ-5D scores were as follows: UK weights, 0.76 (0.74–0.78); US weights, 0.81 (0.80–0.83); and Japan weights, 0.75 (0.73–0.76) (Table 3). The three mean scores were significantly different across methods (ANOVA, *p *< 0.001). Post-hoc Bonferroni tests found that the EQ-5D scores using US weights were significantly higher than those derived from the UK and Japan weights (both, *p *< 0.001). The mean EQ-5D scores using UK and Japan weights, however, did not significantly differ from each other (*p *= 0.68). Medians (interquartile) of the EQ-5D scores from the UK, US, and Japan preference weights were 0.78 (0.69–0.86), 0.83 (0.76–0.85), and 0.73 (0.65–0.79), respectively. The EQ-5D scores using the UK weights yielded the largest range (-0.21–1.00), whereas those scores derived from US and Japan preference weights had similar range (0.06–1.00 and 0.08–1.00, respectively).

**Table 3:** Descriptive statistics of EQ-5D index scores using the UK, US, and Japan preference weights

**Preference weights**	**Mean**	**95% CI**	**Median**	**Q25**	**Q75**	**Range**
UK	0.76	0.74–0.78	0.78	0.69	0.86	-0.21–1.00
US	0.81	0.80–0.83	0.83	0.76	0.85	0.06–1.00
Japan	0.75	0.73–0.76	0.73	0.65	0.79	0.08–1.00

Table 4 presents the estimated mean (95% CI) differences of EQ-5D scores for the UK/US, UK/Japan, and US/Japan schemes were -0.05 (-0.06-(-0.04)), 0.02 (0.01–0.03), and 0.07 (0.06–0.07), respectively. Medians (interquartile) were -0.04 (-0.09–0.00), 0.03 (0.00–0.07), and 0.08 (0.00–0.12) for the UK/US, UK/Japan, and US/Japan weights, respectively. Reported clinically important difference (CID) for the EQ-5D is 0.074 [22]. The median difference between US and Japan weights (0.08) was slightly higher than the CID of 0.074.

**Table 4:** Descriptive statistics of differences in EQ-5D index scores using the UK, US, and Japan preference weights

	**Mean**	**95% CI**	**Median**	**Q25**	**Q75**	**Range**
UK/US	-0.05*	-0.06-(-0.04)	-0.04	-0.09	0.00	-0.35-0.13
UK/Japan	0.02	0.01-0.03	0.03	0.00	0.07	-0.51-0.15
US/Japan	0.07*	0.06-0.07	0.08	0.00	0.12	-0.22-0.13

* *p *< 0.01

### Agreement between the EQ-5D index scores using UK, US, and Japan preference weights

Table 5 illustrates the agreement between EQ-5D values derived from UK, US, and Japan preference weights using ICCs. The ICCs were very high between the pairs of these three approaches, with the highest ICC of 0.97 between UK versus US methods, followed by the ICCs of US versus Japan (0.95) and of UK versus Japan (0.93), respectively.

**Table 5:** Agreement between UK, US, and Japan weights

**Preference weights**	**ICC (95% CI)**
UK & US	0.97** (0.96–0.97)
UK & Japan	0.93** (0.92–0.95)
US & Japan	0.95**(0.94–0.96)

* *p *< 0.01, **p < 0.001

### Bland-Altman Plots

**Figure 2:**
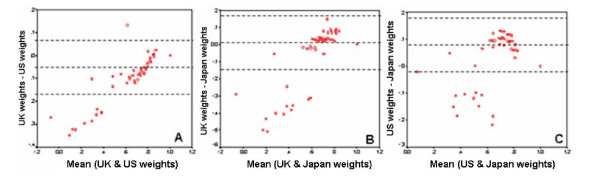
The Bland-Altman plots of EQ-5D scores derived from the UK, US, and Japan preference weights.

Bland-Altman plots were created to compare the agreement among the three EQ-5D scores (Figures 2A–C). These plots showed the differences between scores (Y-axis) and the means of scores (X-axis). The mean of the differences (d) and the limits of agreement were indicated by dotted lines. The 95% limits of agreement were obtained by using the following formula [20]: d ± 1.96*SD of d.

The Bland-Altman plot of UK and US weights showed that 96.4% of the difference scores were between the limits of agreement, 3.3% below the lower agreement line, and 0.3% above the upper agreement line (Figures 2A). Approximately 64% of the UK weights were lower than the US weights (less than zero), 31% are equal, and 5% are higher (greater than zero).

The Bland-Altman plot of UK and Japan weights showed that 96% of the difference scores were between the limits of agreement, 4% below the lower agreement line, and none of the scores above the upper agreement line (Figures 2B). Approximately 64% of the UK weights were higher than the Japan weights (greater than zero), 21% are equal, and 15% are lower (less than zero).

The Bland-Altman plot of US and Japan weights showed that 96.4% of the difference scores were between the limits of agreement, 3.6% below the lower agreement line, and none above the upper agreement line (Figures 2C). Approximately 75% of the US weights were higher than the Japan weights (greater than zero), 21% are equal, and 4% are lower (less than zero).

### Test-retest reliability

The one-two week test-retest reliability of EQ-5D index scores using UK, US, and Japan preference weights (N = 64) is presented in Table 6. It was found that the Japan schemes provided the highest ICCs (0.78) among the three schemes, while the UK and US weights had the same ICCs of 0.74. Rosner suggests that ICC < 0.40 indicates poor agreement, 0.40 ≤ ICC < 0.75 indicates fair to good agreement, and ICC ≥ 0.75 indicates excellent agreement [23]. Based on this criterion, the Japan weights had excellent agreement, whereas both UK and US weights had good agreement on test-retest reliability. It should be noted that in this study the test-retest reliability was conducted via telephone interview whose test-retest correlations were generally lower than those by face-to-face interview [24]. If the test-retest via face-to-face interview had been done, the ICCs of the three approaches should have been increased. Thus, the UK and the US weights might have excellent agreement on test-retest reliability. However, this would not affect the results that the Japan scheme yielded the highest ICC because all three preference weights would have higher ICCs.

**Table 6:** One-two week test-retest reliability of EQ-5D index scores using UK, US, and Japan preference weights (N = 64)

**Preference weights**	**ICC (95% CI)**
UK	0.74** (0.57–0.84)
US	0.74** (0.57–0.84)
Japan	0.78** (0.63–0.86)

* *p *< 0.01, **p < 0.001

### Convergent validity

EQ-5D scores derived from UK, US, and Japan preference weights were significantly associated with all sociodemographic, clinical, and health status variables except for age (Table 7). Spearman's rho correlation coefficients range -0.14 to -0.50. Based on Colton's criteria [25], EQ-5D scores had a little to medium correlation with these variables. However, most magnitudes of correlation between the Japan weighted scores and these variables were higher than those between both UK and US weighted scores and these factors. The magnitudes of correlation between the UK and the US weights and all variables were quite similar.

**Table 7:** Convergent validity of EQ-5D index scores using UK, US, and Japan preference weights

**Variables**	**UK**	**US**	**Japan**
Age (year)	-0.15^a^	-0.02	-0.04
Gender (0 = male, 1 female)	-0.25**	-0.25**	-0.25**
Income (Baht/month)	0.17**	0.17**	0.19**
Duration of diabetes (year)	-0.14*	-0.15*	-0.14*
Body mass index (Kg/m^2^)	-0.15**	-0.15**	-0.19**
HbA1c level (%)	-0.17**	-0.17**	-0.20**
Number of diabetic complications	-0.40**	0.40**	-0.43**
CES-D scores†	-0.49**	-0.49**	-0.50**
VAS scores§	0.46**	0.46**	0.48**

^a ^The number in cells is Spearman's rho correlation coefficients between sociodemographic, clinical, and health status variables.

†Higher scores indicate more depressive symptoms.

§Higher scores indicate better health.

* *p *< 0.05, **p < 0.01

### Known-groups validity

Among the three weighting schemes, the Japan weights obviously showed better discriminant validity than both UK and US weights for all known groups including HbA1c level (above and below 7%) and diabetic complications (presence and absence) namely neuropathy, retinopathy, nephropathy and cardiovascular diseases (Table 8). The relative precision values suggest that the Japan weights discriminated more efficiently than the UK and US weights (the ratios of Japan versus UK and Japan versus US greater than 1.00). Between the UK and US weights, the UK weighted discriminated better for the presence and absence of neuropathy, retinopathy, and cardiovascular complications (the ratios of UK versus US greater than 1.00), whereas, the US weights did more efficiently for HbA1c level and the presence and absence of nephropathy (the ratios of UK versus US less than 1.00).

**Table 8:** Known-groups validity of the EQ-5D index scores using UK, US, and Japan preference weights

	**No.**	**UK**	**US**	**Japan**	**Relative Precision**^a^	**Relative Precision**^b^	**Relative Precision**^c^
HbA1c < 7%	110	0.79	0.83	0.77			
HbA1c ≤ 7%	193	0.75	0.80	0.73			
Difference		0.04	0.03*	0.04*			
Z statistic		-1.91	-2.00	-2.59	0.96	1.36	1.30

Neuropathy (No)	179	0.81	0.84	0.79			
Neuropathy (Yes)	124	0.69	0.77	0.69			
Difference		0.12**	0.07**	0.10**			
Z statistic		-5.94	-5.89	-6.33	1.01	1.06	1.07

Retinopathy (No)	252	0.78	0.82	0.76			
Retinopathy (Yes)	51	0.69	0.76	0.69			
Difference		0.09*	0.06*	0.07**			
Z statistic		-2.16	-2.07	-2.68	1.04	1.24	1.29

Nephropathy (No)	278	0.77	0.82	0.75			
Nephropathy (Yes)	25	0.67	0.75	0.68			
Difference		0.10*	0.07**	0.07**			
Z statistic		-2.57	-2.70	-2.75	0.95	1.07	1.02

Cardiovascular (No)	259	0.78	0.83	0.76			
Cardiovascular Yes)	44	0.65	0.73	0.66			
Difference		0.13**	0.10**	0.10**			
Z statistic		-3.48	-3.45	-3.60	1.01	1.03	1.04

Note: Data in the columns of UK, US, and Japan are mean EQ-5D index scores. The differences in the scores between groups were tested by Mann-Whitney U tests.

* *p*-value < 0.05, **p-value < 0.01

^a ^Ratio of Z statistics for UK weights & US weights

^b ^Ratio of Z statistics for Japan weights & UK weights

^c ^Ratio of Z statistics for Japan weights & US weights

### The impact of the differences in EQ-5D index scores using UK, US, and Japan preference weights on cost-utility analysis

As shown in Table 9, the incremental cost of drug A over drug B was 300,000 Baht for all scenarios. In the base-case scenario, the incremental QALY of using drug A over drug B was 2.4, thus providing an ICUR of 125,000 Baht/QALY. The ICUR for all alternative decision trees ranged from 117,096 Baht/QALY (6.32% difference from the base case) to 123,457 Baht/QALY (1.23% difference from the base case). The seventh decision tree that had the largest percent difference in ICUR from the base case was the scenario using the median difference between US and Japan weights, while the third decision tree that had the smallest percent difference in ICUR from the base case was the scenario employing the mean difference between UK and Japan weights.

**Table 9:** Impact of differences in EQ-5D index scores using UK, US, and Japan preference weights on ICUR for 7 hypothetical decision trees

**Variables**	**Hypothetical decision tree**						
	
	**1**	**2**	**3**	**4**	**5**	**6**	**7**
EQ-5D index scores when the drug was effective (success)							
Drug A	0.900	0.951	0.916	0.967	0.935	0.928	0.981
Drug B	0.800	0.851	0.816	0.867	0.835	0.828	0.881
EQ-5D index scores when the drug was not effective (failure)							
Drug A	0.500	0.551	0.516	0.567	0.535	0.528	0.581
Drug B	0.400	0.451	0.416	0.467	0.435	0.428	0.481
QALY							
Drug A	6.40	6.81	6.53	6.94	6.68	6.62	7.05
Drug B	4.00	4.31	4.10	4.40	4.21	4.17	4.49
Incremental cost (Baht)	300,000	300,000	300,000	300,000	300,000	300,000	300,000
Incremental QALY	2.40	2.50	2.43	2.54	2.47	2.45	2.56
ICUR (Baht/QALY)	125,000	119,904	123,457	118,110	121,457	122,150	117,096
% Difference in ICUR from base case (Decision tree 1)	-	4.08	1.23	5.51	2.83	2.28	6.32

Decision tree 1 represents the base-case scenario, while decision trees 2–7 mean the scenarios where base-case utility scores overestimated by 0.051 (mean difference between UK versus US weights), 0.016 (mean difference between UK versus Japan weights), 0.067 (mean difference between US versus Japan weights), 0.035 (median difference between UK versus US weights), 0.028 (median difference between UK versus Japan weights), and 0.081 (median difference between US versus Japan weights).

## Discussion

To the best of our knowledge, this is the first study examining the differences and cross-cultural validation between EQ-5D scores derived from UK, US, and Japan preference weights. The results showed that there were significant differences across the three EQ-5D index scores. US weights yielded higher scores than those of UK and Japan weights (*p *< 0.001, both), while the UK and Japan weighted scores did not differ (*p *> 0.05). The EQ-5D index scores derived from both UK and Japan weights were also comparable to that of a previous study which showed that type 2 diabetes provided the mean EQ-5D score of 0.75 [26]. Both UK and US scores had more agreement with each other than with the Japan scores. As for psychometric properties, the Japan scheme provided better test-retest reliability, convergent and known-groups validity than both UK and US schemes. We also determined the impact of the differences in these EQ-5D index scores on the outcome of CUA. It was found that variation in utility scores estimated from UK, US, and Japan preference weights had a relatively small impact on CUA (range: 1.23–6.32%).

Our study showed that the US weighted scores were higher than the UK weighted scores. This result is consistent with the previous study conducted in US patients living with HIV infection [17]. However, our study yielded larger mean difference scores (mean difference = 0.05) than those of the previous study (mean difference = 0.03). This may be due to differences in health states of patient populations. Johnson et al found that the discrepancy between the US and UK schemes was smaller for better health states, but larger for extreme health problems [15]. This finding is also similar to our study (please see Figure 2A). In the previous US study, the HIV patients had better health (mean EQ-5D scores using US and UK was 0.87 and 0.84, respectively) than those of the diabetic patients in the present study (mean EQ-5D scores using US and UK was 0.81 and 0.76, respectively). Therefore, this may be the reason why the larger mean difference between US and UK was found in the present study.

This study also showed that the EQ-5D index scores using the US scheme were higher than those of the Japan scheme with the estimated mean difference of 0.07, while the UK model yielded slightly higher scores than the Japan model with the mean difference of 0.02 (not statistically significant). No previous study has compared between US and Japan weighted scores; however, the large discrepancy may be attributable to differences in algorithms, cultures, research methods, and/or other factors. Tsuchiya and colleagues have reported that the Japan scheme yielded consistently higher scores than the UK weights except for the very mild states [11]. This finding contrasted with our results that the mean UK weighted scores had slightly higher than the mean Japan index scores but they were not significantly different. Also, the Bland-Altman plot (Figure 2B) presented that the majority of the UK weighted scores (62%) was higher than the Japan weights except for the extreme health states. These different results may be due to the fact that they did the previous study in a general population, but we used a real patient population. The utility weights derived from a heterogeneous general population and applied to a patient population may be less precise to detect differences across cultures. In addition, due to differences in population ratings and healthcare settings between Japan and Thailand, EQ-5D valuations would perform differently when applied to different populations.

It is not surprising that UK and US preference weights had more agreement with each other than with Japan weights because they are western countries whose cultures are different from that of Japan which is in Asia. Moreover, the Japan scheme provided better test-retest reliability, convergent and known-groups validity than both UK and US schemes in this Thai sample. These results may reflect the fact that Thailand is an Asian country whose culture is closer to Japan than to both UK and US. Thus, it is more likely that the Japan weights should be used for EQ-5D valuations for Thai people than the UK or US weights.

Even though our results showed that there was difference in EQ-5D scores derived from the UK, US and Japan weights, the impact on ICUR was marginal. This leads to the question of which preference weights should be used and in what situations. All of our results suggest that if the EQ-5D index scores is used as a HRQoL measure for the purpose of clinical decision making such as using the utility scores to be a clinical indicator to monitor patients' health status, the Japan should be applied for Thais. However, if one would like to evaluate CUA or CEA whose outcomes are QALYs gained, the choice of weighting scheme does not matter. Nevertheless, if we have to recommend a method, the Japan should be the most appropriate one because they demonstrated better psychometric properties than the UK and US weights.

The results of this study need to be interpreted in the light of these following limitations. First, we used only cross-sectional data. Differences in change scores may be likely to have a greater impact on ICUR than changes in absolute scores. Thus, further study should be done in longitudinal data. Second, our data were derived from diabetic outpatients, so the results were limited to a specific patient group. The findings are not likely to be able to be generalized to other patient populations. Other clinical populations need investigation. Finally, we utilized a simple hypothetical decision tree model to examine the impact of variability in EQ-5D index scores on ICUR. Therefore, using real CUA data should be more informative.

## Conclusion

In this study, we compared weights on EQ-5D valuations using algorithms developed in the UK, US, and Japan general populations, but cross-validated using a Thai patient sample. Our results suggest that the US scheme provided higher EQ-5D index scores than the UK and Japan schemes, while the UK and Japan weighted scores did not significantly differ. However, the impact of the differences in these EQ-5D index scores on the outcome of CUA was quite small. Both UK and US scores had more agreement with each other than with Japan scores. The Japan scheme provided better test-retest reliability, convergent and known-groups validity than both UK and US schemes. We recommended that among these three weights the Japan model should be used in Thai people. However, more research needs to be done.

## Abbreviations

HU: health utility; HRQoL: health-related quality of life; CUA: cost-utility analyses; QALYs: quality-adjusted life-years; EQ-5D: EuroQoL; VAS: visual analog scale; UK: United Kingdom; US: United States; CES-D: Center for Epidemiologic Studies Depression; TTO: time trade-off; ANOVA: analysis of variance; ICCs: intraclass correlations coefficients; SD: standard deviation; 95% CI: 95% confidence interval; CID: clinically important difference; ICUR: incremental cost-utility ratio.

## Competing interests

The authors declare that they have no competing interests.

## Authors' contributions

PS was responsible for the conception of the study, analyzing the data, and writing the article. RC contributed to analyzing the data and the interpretation of the results. RS contributed to analyzing and collecting the data. All authors have read and approved the final manuscript.
